# ^18^F-florbetapir PET/MRI for quantitatively monitoring demyelination and remyelination in acute disseminated encephalomyelitis

**DOI:** 10.1186/s13550-019-0568-8

**Published:** 2019-11-12

**Authors:** Min Zhang, Jun Liu, Biao Li, Sheng Chen

**Affiliations:** 1 Department of Nuclear Medicine, Ruijin Hospital, Shanghai Jiao Tong University School of Medicine, Shanghai, China; 2 Department of Neurology, Ruijin Hospital, Shanghai Jiao Tong University School of Medicine, 197 Ruijin 2nd Road, Shanghai, 200025 China

Dear Editor,

Acute disseminated encephalomyelitis (ADEM) is one of the most important inflammatory demyelinating disorders of the central nervous system [1]. Magnetic resonance imaging (MRI) is essential for the diagnosis and follow-up of demyelinating disease. However, conventional MRI measurement cannot provide a direct quantitative assessment of demyelination and remyelination [2]. Advanced MRI techniques such as magnetization transfer imaging [3] or myelin water fraction [4] are increasingly popular as research tools but have not yet been standardized for widespread clinical application.

Positron emission tomography (PET) is a noninvasive technique for quantitative imaging of biochemical and physiological processes. Amyloid PET tracer ^11^C-PiB has been studied for the quantitative assessment of myelin content in preclinical [5, 6] and clinical studies [7–10]. However, the short half-life of carbon-11 limits the use of ^11^C-PiB in clinical practice. Therefore, some fluorine-18-labeled amyloid tracers including ^18^F-florbetaben [11, 12] and ^18^F-florbetapir [13] have been investigated as imaging marker for quantification of myelin loss in patients with multiple sclerosis (MS). Our recent study also showed that myelin histology correlated quantitatively with ^18^F-florbetapir binding in demyelinated lesions [14]. Nevertheless, the ability of fluorine-18-labeled amyloid tracers for assessing remyelination has not yet been demonstrated. In addition, as MRI is more sensitive than computed tomography (CT) in the detection of demyelinated lesion, a hybrid PET/MRI can obtain more reliable semi-quantitative measurements of tracer uptake than PET/CT. Consequently, we for the first time, to our knowledge, present a case of hybrid PET/MRI with ^18^F-florbetapir for quantitatively monitoring demyelination and remyelination in a 59-year-old female patient with ADEM.

The patient admitted to our hospital because of acute onset of confusion, impaired short-term memory, muscle weakness, and positive Babinski sign on the left side for 4 days. She has no similar attack in the past. She had upper respiratory infection history 1 week prior her symptoms started. Her mini-mental state examination (MMSE) score was 18, and expanded disability status scale (EDSS) score was 7. On admission, cerebral spinal fluid (CSF) examination indicated protein elevation and lymphocytic pleocytosis. Oligoclonal band was negative in CSF. Serum and CSF antibodies against aquaporin 4 (AQP4) and myelin oligodendrocyte glycoprotein (MOG) were all negative. T1-weighted MRI with gadolinium showed disseminated subcortical lesions with marginal mild enhancement and central “black hole” (Additional file 1), suggesting destruction of blood-brain barrier and axonal loss. 

On pre-treatment PET/MRI, T2 FLAIR image demonstrated multifocal hyperintense lesions (Fig. 1a, c, e, white arrow) in damaged white matter (DWM). Registered to T2 FLAIR image, the volume of interest in the largest five DWM lesions was manually delineated on PET images, and the cerebellum was used as the reference region for the standardized uptake value relative ratios (SUVR) (Additional file 1). We found that the ^18^F-florbetapir binding (Fig. 1b, d, f) in DWM (mean SUVR = 0.74 ± 0.04) was significantly lower than that in normal-appearing white matter (NAWM) (mean SUVR = 0.97 ± 0.03) indicating a myelin loss in DWM.

**Fig. 1:**
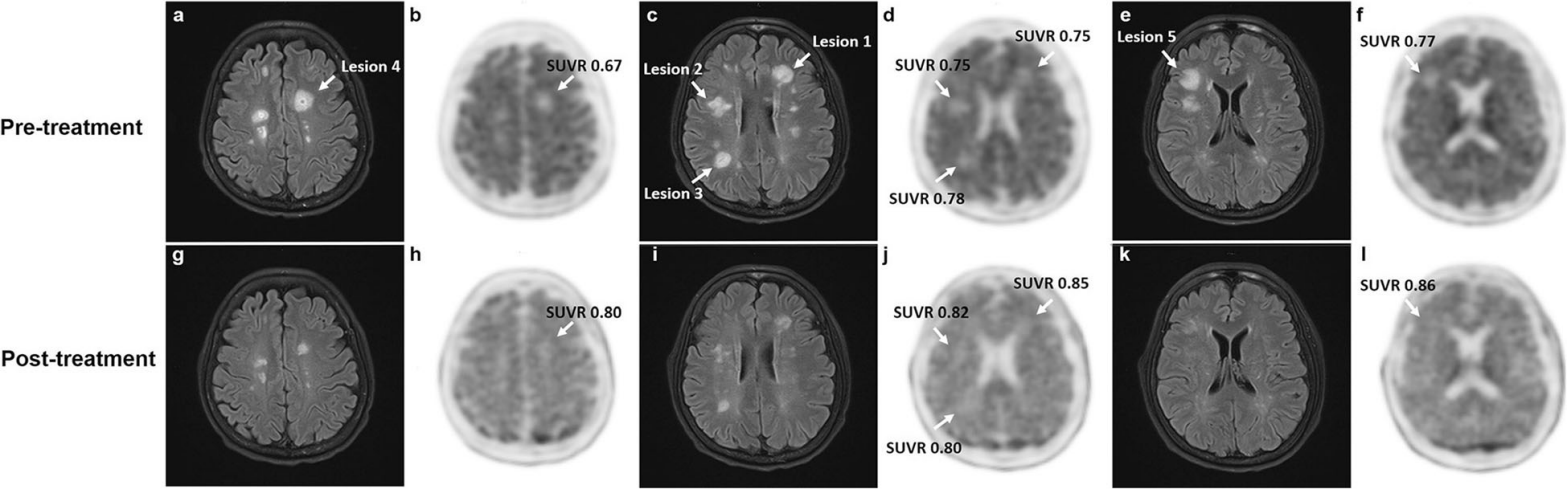
Demyelination and remyelination in five representative lesions on pre- and post-treatment ^18^F-florbetapir PET/MRI. **a**–**f** Pre-treatment PET/MRI showed multifocal hyperintense lesions (white arrow) with decreased ^18^F-florbetapir uptake (SUVR = 0.75, 0.75, 0.78, 0.67, and 0.77). **g**–**l** Five months later, reduced hyperintense lesions with increased ^18^F-florbetapir uptake (SUVR = 0.85, 0.82, 0.80, 0.80 and 0.86) were observed on post-treatment PET/MRI

Based on her medical history, clinical manifestations and PET/MRI findings, the diagnosis of ADEM was made. The patient then received the course of intravenous immunoglobulin at 0.4 g/kg/day for 5 days and intravenous methylprednisolone 500 mg/day for 5 days followed by a tapering dose of prednisone. Five months later, she had normal muscle strength on the left side. Her MMSE score returned to 29, and EDSS score improved to 1. Repeated lumbar puncture showed normal cell count and protein level. Consistent with the improvements in clinical symptoms and scoring, post-treatment PET/MRI showed a reduction of the hyperintense lesions (Fig. 1g, i, k) and an increased but still lower than normal ^18^F-florbetapir uptake (Fig. 1h, j, l) in DWM (mean SUVR = 0.82 ± 0.03) reflecting an incomplete remyelination.

With this letter, we would like to suggest that ^18^F-florbetapir PET as an additional supplement to conventional MRI has good potential for quantitative assessment of the progression of demyelinating disease such as ADEM, MS, and neuromyelitis optica, and it may be considered in the future for evaluation of efficacy of new treatments which promote remyelination [15]. However, the sensitivity and specificity of ^18^F-florbetapir PET/MRI needs to be further evaluated in a larger group of patients.

## Supplementary information

**Additional file 1:** **Figure S1.** T1 weighted MR with gadolinium in the patient with ADEM before treatment. It showed disseminated subcortical lesions (white arrow) with marginal mild enhancement and central “black hole”, suggesting destruction of blood-brain barrier and axonal loss. **Table S1.** SUVR of the VOIs in the representative DWMs, NAWMs and right cerebellum. **Figure S2.** Change of SUVR in the five DWM lesions between pre-treatment and post-treatment.

## Data Availability

All data generated or analyzed during this study are included in this published article [and its supplementary information files].
