# Supplementary material for: 18F-florbetapir PET/MRI for quantitatively monitoring demyelination and remyelination in acute disseminated encephalomyelitis
Source: EJNMMI Res. 2019 Nov 12;9:96. doi: 10.1186/s13550-019-0568-8 (PMC6851275; doi:10.1186/s13550-019-0568-8)
Supplement: Supplementary file 1 — Additional file 1: Figure S1. T1 weighted MR with gadolinium in the patient with ADEM before treatment. It showed disseminated subcortical lesions (white arrow) with marginal mild enhancement and central “black hole”, suggesting destruction of blood-brain barrier and axonal loss. Table S1. SUVR of the VOIs in the representative DWMs, NAWMs and right cerebellum. Figure S2. Change of SUVR in the five DWM lesions between pre-treatment and post-treatment. [file 13550_2019_568_MOESM1_ESM.docx]

Materials and methods

A hybrid PET/MRI scan was performed with Biograph mMR system (Siemens, Erlangen, Germany). After intravenous injection of 296 MBq of ^18^F-florbetapir in the patient, dynamic PET acquisition in list mode over 60 min was started immediately. During PET acquisition, a 3D T1 magnetization-prepared rapid acquisition gradient echo (T1 MPRAGE, Repetition Time 1900 ms; Echo Time 2.44 ms; slice thickness 1mm) and a 3D T2-weighted fluid-attenuated inversion recovery (T2 FLAIR, Repetition Time 5000 ms; Echo Time 385 ms; slice thickness 0.9 mm) were acquired. PET image was reconstructed by point spread function algorithm with 344 × 344 pixels, 4 iterations, 21 subsets and a filter with full width at half maximum of 2 mm, and then one frame of all 60 min in the form of transaxial images was finally obtained.

PET analysis was performed using the software Siemens syngo^®^. Registered to T2 FLAIR image, a spherical volume of interest (VOI) on the largest five DWMs, three representative NAWM regions and right cerebellum was manually delineated on the pre-treatment PET images, and then these VOIs were copied onto the same slices of post-treatment PET images. The mean standardized uptake value (SUV_mean_) of all VOIs was automatically measured. The right cerebellum encompassing grey and white matter was used as the reference region for the calculation of SUV relative ratio (SUVR).

Figure S1. T1 weighted MR with gadolinium in the patient with ADEM before treatment. It showed disseminated subcortical lesions (white arrow) with marginal mild enhancement and central “black hole”, suggesting destruction of blood-brain barrier and axonal loss.


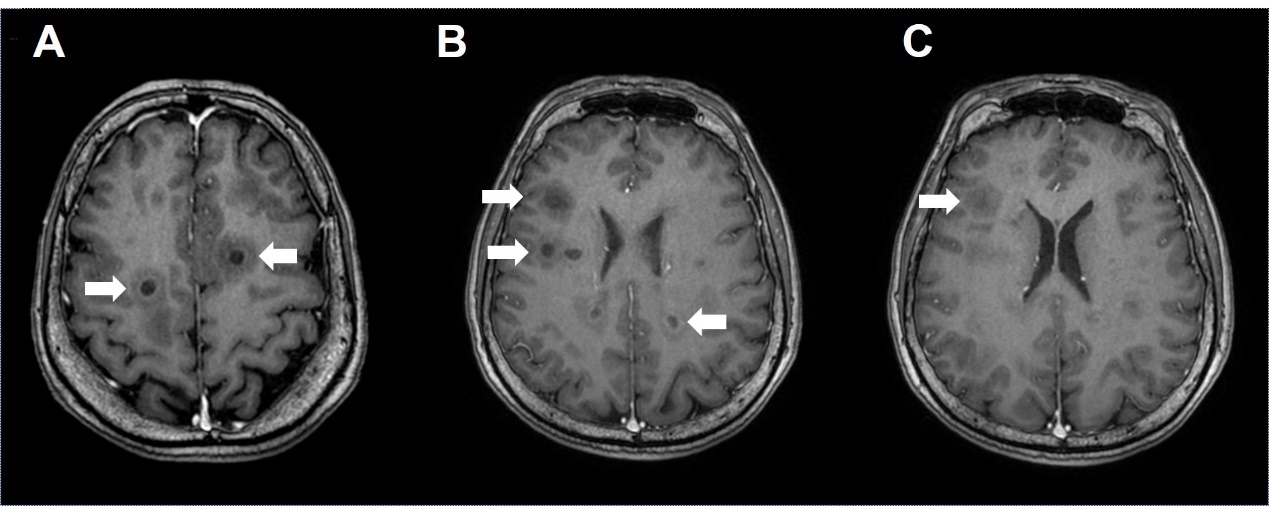


Table S1. SUVR of the VOIs in the representative DWMs, NAWMs and right cerebellum.

|  | VOI | Pre-treatment | | Post-treatment | |
| --- | --- | --- | --- | --- | --- |
|  | (cm^3^) | SUV_mean_ | SUVR | SUV_mean_ | SUVR |
| DWM Lesion 1 | 1.46 | 1.09 | 0.75 | 1.04 | 0.85 |
| DWM Lesion 2 | 1.25 | 1.09 | 0.75 | 1.00 | 0.82 |
| DWM Lesion 3 | 1.10 | 1.13 | 0.78 | 0.97 | 0.80 |
| DWM Lesion 4 | 1.43 | 0.97 | 0.67 | 0.97 | 0.80 |
| DWM Lesion 5 | 1.87 | 1.12 | 0.77 | 1.05 | 0.86 |
| NAWM in right temporal lobe | 2.11 | 1.45 | 1.00 | 1.15 | 0.94 |
| NAWM in left temporal lobe | 2.10 | 1.36 | 0.94 | 1.10 | 0.90 |
| NAWM in left frontal lobe | 1.20 | 1.40 | 0.97 | 1.12 | 0.92 |
| Right cerebellum | 1.38 | 1.45 | / | 1.22 | / |

SUVR: standardized uptake value relative ratios

DWM: damaged white matter

NAWM: normal-appearing white matter

VOI: volume of interest

Figure S2. Change of SUVR in the five DWM lesions between pre-treatment and post-treatment
